# Statin Use and the Risks of Delirium and Short-Term Mortality in Hospitalized Adults: An Updated Systematic Review and Meta-Analysis

**DOI:** 10.3390/biomedicines14102291

**Published:** 2026-10-09

**Authors:** Tzu-Rong Peng, Ta-Wei Wu, Yun-Hui Huang, Shih-Ming Chen, Ming-Chia Lee

**Affiliations:** 1 Department of Pharmacy, Taipei Tzu Chi Hospital, Buddhist Tzu Chi Medical Foundation, New Taipei City 23142, Taiwan; tzu.rong@tzuchi.com.tw (T.-R.P.); tawei@tzuchi.com.tw (T.-W.W.); b405101056@gmail.com (Y.-H.H.); 2 School of Pharmacy, College of Pharmacy, Taipei Medical University, Taipei 11031, Taiwan; smchen@tmu.edu.tw; 3 Department of Pharmacy, New Taipei City Hospital, New Taipei City 24141, Taiwan

**Keywords:** statins, delirium, meta-analysis

## Abstract

**Background/Objectives**: Delirium is a common and serious neuropsychiatric complication in hospitalized and critically ill patients and is associated with prolonged hospitalization and increased mortality. Previous meta-analyses found no clear association between statin use and delirium; however, newly published studies and emerging mortality data warrant re-evaluation. **Methods**: This updated systematic review and meta-analysis extended a previous meta-analysis and re-evaluated the association between statin use and delirium risk. PubMed, Embase, and the Cochrane Library were searched for eligible studies published from January 2022 through October 2025. Study-level estimates were synthesized using random-effects models. Because ORs, RRs, and HRs are not directly interchangeable, the combined synthesis was considered exploratory. Sensitivity analyses included an OR-only dataset, exclusion of the cross-sectional study, REML models with Hartung–Knapp adjustment, and restriction to observational studies reporting multivariable-adjusted ORs. **Results**: Nineteen studies (5 randomized controlled trials and 14 observational studies) involving 440,737 participants were included. The exploratory combined analysis yielded an OR of 0.79 (95% confidence interval [CI] 0.66–0.95; *p* = 0.01; I2 = 90%). Results differed by study design: randomized trials yielded a lower pooled point estimate (OR 0.41, 95% CI 0.20–0.82; I2 = 58%), whereas the pooled association among observational studies was not statistically significant (OR 0.86, 95% CI 0.71–1.03; I2 = 91%; *p* for subgroup difference = 0.04). However, in the three randomized trials contributing to incident delirium, REML with Hartung–Knapp adjustment substantially widened the confidence interval (OR 0.41, 95% CI 0.09–1.86; *p* = 0.126). In the overall analysis, REML–Hartung–Knapp inference also increased uncertainty (OR 0.79, 95% CI 0.62–1.00; *p* = 0.047), as did restriction to multivariable-adjusted observational ORs (OR 0.76, 95% CI 0.57–1.01; *p* = 0.062). In four studies comprising 21,687 participants, statin use was associated with lower 30-day all-cause mortality under the conventional random-effects model (OR 0.52, 95% CI 0.35–0.79; *p* = 0.002; I2 = 88%); this estimate remained similar under REML with Hartung–Knapp adjustment (OR 0.52, 95% CI 0.32–0.84; *p* = 0.022). No significant association was observed for delirium-free days. **Conclusions**: The available evidence suggests a possible association between statin exposure and lower delirium occurrence, but substantial heterogeneity, differences between randomized and observational evidence, and sensitivity to analytic assumptions limit causal interpretation. The findings do not establish statins as a preventive treatment for delirium or prove a mortality benefit. Further adequately powered randomized trials with clearly defined exposure timing and standardized delirium assessment are warranted.

## 1. Introduction

Delirium is an acute disturbance of attention, awareness, and cognition and is characterized by a fluctuating course and rapid onset. Among hospitalized patients, especially those in an intensive care unit (ICU), postoperative setting, or geriatric ward, delirium is far from rare. A meta-analysis of 48 ICU studies involving 27,342 patients estimated that the prevalence of delirium is 31% [95% confidence interval (CI) 24–41%] in adult ICU populations [1]. The clinical consequences of delirium are profound. Patients in an ICU who develop delirium tend to require mechanical ventilation for longer periods, have longer ICU and hospital stays, and have an increased risk of complications [2]. Furthermore, delirium is associated with increased mortality risk. A systematic review conducted on general hospital populations observed a substantially higher all-cause mortality rate among patients with delirium than among those without [3]. In addition to increasing morbidity and mortality risks, delirium imposes a heavy economic burden on health-care systems. A systematic review of delirium-associated costs reported that the adjusted per-patient cost ranges from US$806 to US$24,509 (in terms of USD for 2019) depending on the research setting and methodology [4].

Delirium is caused by converging disturbances in neuroinflammatory signaling, oxidative stress, cholinergic dysregulation, other neurotransmitter dysregulation, microcirculatory and endothelial dysfunction, and blood–brain barrier (BBB) disruption. A 2020 review reported that systemic inflammation and BBB disruption are the central mechanisms underlying delirium, particularly in surgical and critically ill patients, and associated with peripheral immune activation in response to altered brain network connectivity and acute cognitive failure [5]. Although statins are primarily prescribed to protect the cardiovascular system and reduce lipid levels, they possess pleiotropic properties, including anti-inflammatory, antioxidative, and endothelial-protective effects, that may confer neuroprotection [6]. The underlying mechanisms are well described in a translational review examining the effects of statins on the neurovascular unit [7].

Two previous meta-analyses did not identify a clear association between statin use and delirium. In 2017, Vallabhajosyula and colleagues, who pooled six studies involving 289,773 patients, observed no reduction in the likelihood of delirium with statin use overall [relative risk (RR) 1.05, 95% CI 0.85–1.29] or in cardiac surgery cohorts, and they detected substantial heterogeneity [8]. In 2023, Chang et al. reviewed evidence obtained up to December 2021 and again determined a nonsignificant association between the use of statins and risk of delirium [pooled odds ratio (OR) 0.82, 95% CI 0.64–1.04] [9]. Since 2022, an increasing number of studies have examined the association between statin therapy and delirium risk, but their results have again been inconsistent. Several recent studies have reported an association between statin use and a lower risk of delirium [10,11,12,13], whereas other studies have reported null or opposite associations [14]. These conflicting findings highlight persistent uncertainty regarding the direction and magnitude of the association between statin exposure and delirium. Accordingly, an updated systematic review and meta-analysis is warranted to clarify the available evidence and to assess the consistency and robustness of the association across different study designs and clinical settings.

## 2. Materials and Methods

### 2.1. Study Design and Reporting Framework

This updated systematic review and meta-analysis re-evaluated the association between statin use and delirium risk across diverse clinical populations. The review followed the Preferred Reporting Items for Systematic Reviews and Meta-Analyses (PRISMA 2020) guidelines and adhered to established methodological standards for evidence synthesis [15]. The PRISMA checklist is provided in Table S1. The protocol was prospectively registered with the International Prospective Register of Systematic Reviews (PROSPERO; CRD420251171077).

### 2.2. Literature Search Strategy

The present study is a continuation of our previous meta-analysis that comprehensively reviewed articles published up to 31 December 2021 [9]. To extend the evidence base, we performed a new systematic search covering the period from 1 January 2022 to 20 October 2025. To ensure continuity and comparability, the updated search had the same methodological framework and employed the same inclusion criteria as the original review. We systematically searched PubMed, Embase, and the Cochrane Library for relevant studies by using both controlled vocabulary (MeSH/Emtree) and free-text terms related to statin therapy and delirium (listed in Box A1). No language or geographic restrictions were applied. The reference lists of included studies and relevant reviews were also manually searched to identify additional potentially eligible studies. The complete database-specific search strategies are provided in Appendix A exactly as executed, including the search dates and applied limits. Trial registries, preprint servers, grey-literature databases, and citation-index databases were not searched; therefore, unpublished or non-indexed studies may have been missed. All retrieved records were imported into EndNote 20, which was used to remove duplicates. Two reviewers (M.C.L. and T.R.P.) then independently screened the records. Disagreements were resolved through consensus or adjudication by a third author (S.M.C.).

### 2.3. Eligibility Criteria

Studies were eligible for inclusion if they were a randomized controlled trial (RCT) or observational study that provided comparative data on the use of statins and occurrence of delirium. The eligible populations were adult patients (those aged 18 years or older) who were admitted to a medical unit, a surgical unit, or an ICU. The exposure of interest was statin therapy—initiated before admission, perioperatively, or during hospitalization—regardless of the specific statin type or dosage. The comparator group consisted of patients who did not receive a statin or who received placebo. The primary outcome was the incidence or risk of delirium, as diagnosed using a validated assessment tool or standardized diagnostic criteria, such as the Confusion Assessment Method (CAM), CAM for the ICU (CAM-ICU), Diagnostic and Statistical Manual of Mental Disorders codes, or International Classification of Diseases (ICD) codes. The secondary outcome was 30-day all-cause mortality, as reported in studies that provided comparable short-term survival data. Mortality was selected as a secondary endpoint because in hospitalized and critically ill patients, delirium is strongly associated with adverse outcomes and an increased short-term risk of mortality. In addition, we evaluated the number of delirium-free days as an exploratory outcome; this number reflects the duration of time a patient remained free from delirium during follow-up. Studies enrolling patients with established delirium were eligible only for analyses of delirium duration or delirium-free days and were not included in the incident-delirium synthesis. Studies were required to report quantitative effect estimates, including ORs, risk ratios (RRs), or hazard ratios (HRs) with corresponding 95% CIs. Conference abstracts, narrative reviews, case reports, pediatric studies, and articles lacking sufficient statistical information to calculate effect estimates were excluded.

### 2.4. Study Selection and Data Extraction

Two independent reviewers (M.C.L. and T.R.P.) screened eligible studies in a two-step process consisting of initial title and abstract screening followed by full-text review. Discrepancies were resolved through consensus or adjudication by a third author (S.M.C.). Data were extracted from articles by using a prespecified standardized form that captured the following: study characteristics (first author, publication year, country, design, setting, and sample size), population demographics, delirium diagnostic tool, definition and timing of statin exposure, number of delirium events, and crude and adjusted effect estimates with 95% CIs, along with the covariates used for adjustment. When multiple multivariable models were presented, the model with the most complete adjustment was selected.

### 2.5. Quality Assessment

The methodological quality of included observational studies was determined using the Newcastle–Ottawa Scale [16], which comprises selection, comparability, and outcome domains and has a maximum score of 9. The RCTs were evaluated using the Cochrane Risk of Bias 2.0 tool [17]. Two reviewers (M.C.L. and T.R.P.) independently performed the quality assessment, and any discrepancies were resolved through discussion with a third author (S.M.C.).

### 2.6. Statistical Analysis

Effect estimates were synthesized using the generic inverse-variance method after logarithmic transformation. Adjusted estimates were preferentially selected when available. Because odds ratios (ORs), risk ratios (RRs), and hazard ratios (HRs) are not directly interchangeable, particularly when outcome incidence is substantial, the combined synthesis of different relative effect measures was regarded as exploratory. A sensitivity analysis restricted to studies reporting ORs or providing sufficient binary data for calculation of ORs was therefore performed, with studies reporting only an RR or HR excluded from this OR-only analysis.

Random-effects models using the DerSimonian–Laird method were used for the primary analyses. Given the substantial between-study heterogeneity, robustness was additionally examined using restricted maximum likelihood (REML) estimation with Hartung–Knapp adjustment. Statistical heterogeneity was assessed using Cochran’s Q test and quantified using the I^2^ statistic, with values of approximately 25%, 50%, and 75% interpreted as low, moderate, and high heterogeneity, respectively. To further characterize between-study variability, a 95% prediction interval was calculated under the REML random-effects model to estimate the range of true effects that might be expected in a future study conducted under similar conditions.

Prespecified subgroup analyses explored potential sources of heterogeneity according to (1) study-level mean or median age (<60 vs. ≥60 years), (2) delirium ascertainment method (CAM-ICU vs. CAM or ICD codes), (3) geographic region (Asian vs. non-Asian countries), and (4) study design. Because randomized and observational studies differ in exposure allocation and susceptibility to confounding, the study-design subgroup was considered particularly important. Age- and geography-based subgroup analyses were considered ecological and hypothesis-generating.

Sensitivity analyses included sequential omission of each study, exclusion of the cross-sectional study, an OR-only analysis, and a stricter observational analysis restricted to studies explicitly reporting multivariable-adjusted ORs with sufficient information on covariate adjustment. Because only a small number of randomized trials and studies reporting 30-day mortality were available, additional sensitivity analyses were performed for these subsets using REML estimation with Hartung–Knapp adjustment, and 95% prediction intervals were calculated to characterize uncertainty in the expected effect across comparable settings. In addition, an exploratory subgroup analysis according to statin exposure timing was conducted among OR-reporting studies for which exposure timing could be clearly classified. Studies were categorized as evaluating pre-existing, preadmission, or preoperative statin use versus newly initiated or protocol-assigned statin therapy. Studies with mixed or insufficiently reported exposure timing were excluded from this analysis. Differences between exposure-timing subgroups were evaluated using a mixed-effects meta-regression model with REML estimation and Hartung–Knapp adjustment. These analyses were performed to assess the influence of effect-measure choice, study design, confounding control, exposure timing, and random-effects assumptions on the pooled estimates.

Potential publication bias was evaluated by visual inspection of funnel plots and by Begg’s rank correlation test and Egger’s regression test. In addition, Duval and Tweedie’s trim-and-fill procedure was applied when at least 10 studies contributed to the outcome of interest. All statistical analyses were performed using STATA version 17.0 (StataCorp, College Station, TX, USA), Review Manager version 5.4 (The Cochrane Collaboration, London, UK), or R with the metafor package for REML, Hartung–Knapp, prediction-interval, and mixed-effects meta-regression analyses.

The certainty of evidence for each outcome (delirium incidence, 30-day all-cause mortality, and delirium-free days) was evaluated using the Grading of Recommendations Assessment, Development and Evaluation (GRADE) approach. This framework considers five domains—risk of bias, inconsistency, indirectness, imprecision, and publication bias—and rates the certainty of evidence as high, moderate, low, or very low. Two reviewers (M.C.L. and T.R.P.) independently assessed each domain, and disagreements were resolved through discussion with a third author (S.M.C.).

## 3. Results

### 3.1. Search Results and Study Characteristics

**Figure 1:**
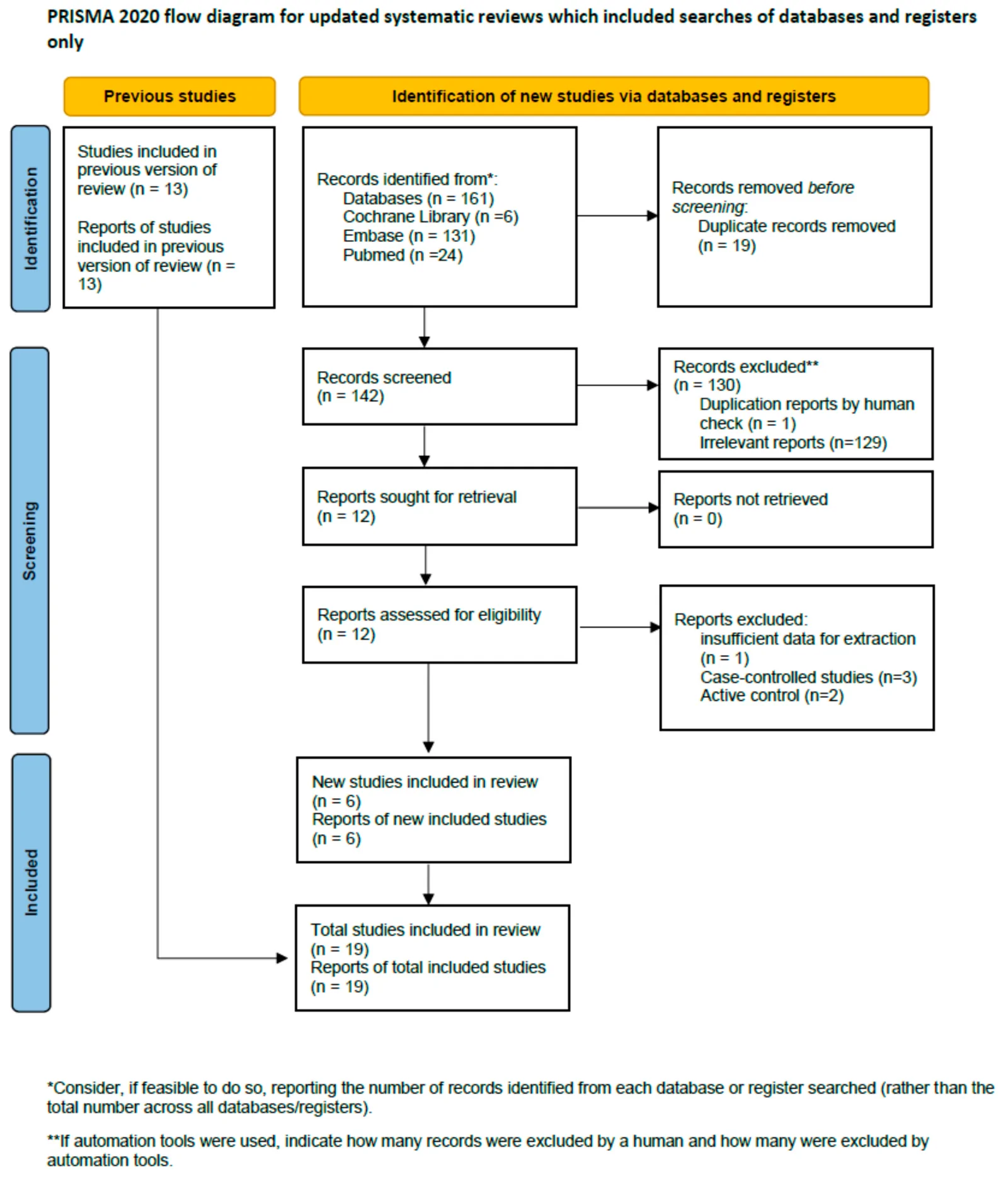
Flowchart of the literature search process.

A total of 161 records were identified in PubMed (*n* = 24), Embase (*n* = 131), and the Cochrane Library (*n* = 6). Of these, 19 duplicate records were removed, leaving 142 titles and abstracts to be screened for eligibility. Of the 142 records, 130 were excluded (129 were irrelevant and 1 was a duplicate identified through manual inspection). The full texts of the remaining 12 reports were assessed for eligibility, but 6 were excluded for the following reasons: insufficient data for extraction (*n* = 1), a case–control design (*n* = 3), and use of an active comparator instead of a non-statin-user control (*n* = 2). Ultimately, 6 new studies met the inclusion criteria and were incorporated into the quantitative synthesis, in addition to the 13 studies included in the previous version of the review [9]. Thus, 19 studies were included in the updated meta-analysis (Figure 1). The characteristics of these 19 studies are summarized in Table 1. In total, 440,737 participants from nine countries were included in the analysis. The mean participant age ranged from 51.0 to 79.9 years in the individual studies.

### 3.2. Quality Assessment

The risk-of-bias evaluation of the five RCTs is presented in Figure S1. Four RCTs [11,24,26,30] were judged to have a low risk of bias across all domains, whereas the trial by Sohrevardi et al. [29] raised some concerns regarding the randomization process because sequence generation was insufficiently described. The quality assessment of the 14 observational studies [10,12,13,14,18,19,20,21,22,23,25,27,28,31] is summarized in Table S2. Newcastle–Ottawa Scale scores ranged from 6 to 9; however, the extent and quality of confounder adjustment varied substantially across studies (Table S3).

### 3.3. Meta-Analysis and Sensitivity Analysis

**Figure 2:**
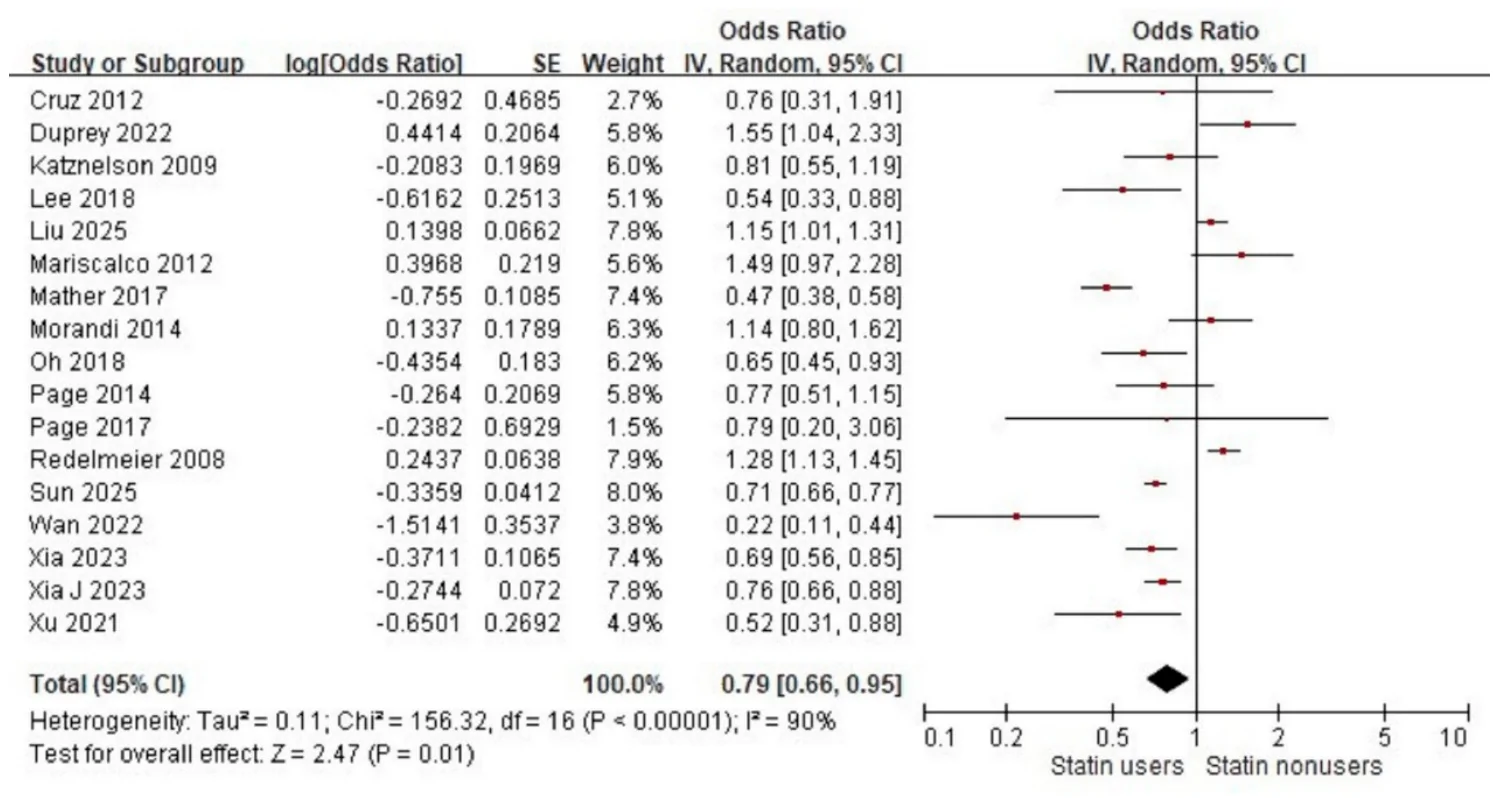
Forest plot of the summary odds ratios for the association between statin use and delirium [10,11,12,13,14,18,19,20,21,22,23,25,26,27,28,30,31].

In the exploratory synthesis combining randomized and observational evidence, statin use was associated with a lower summary OR for delirium (OR 0.79, 95% CI 0.66–0.95; *p* = 0.01; I^2^ = 90%; Figure 2). The substantial heterogeneity indicated considerable variation in study-level effects. Leave-one-out analyses produced broadly similar point estimates, although statistical significance became borderline after exclusion of Wan et al., and substantial heterogeneity persisted throughout the analyses (Table S4). Several additional sensitivity analyses were performed (Table S5; Figure S5). Restricting the analysis to ORs yielded a similar estimate (OR 0.76, 95% CI 0.61–0.94; *p* = 0.013; I^2^ = 89%). Excluding the cross-sectional study by Liu et al. also produced a similar estimate (OR 0.77, 95% CI 0.64–0.93; *p* = 0.007; I^2^ = 88%). Under REML with Hartung–Knapp adjustment, the pooled estimate remained similar, but the confidence interval widened and statistical significance became marginal (OR 0.79, 95% CI 0.62–1.00; *p* = 0.047; I^2^ = 92.7%; τ^2^ = 0.154). The corresponding 95% prediction interval was 0.33–1.87, spanning the null value and indicating substantial between-study variability in the expected effect across settings. In a stricter analysis restricted to seven observational studies explicitly reporting multivariable-adjusted ORs, the pooled estimate remained below unity but was not statistically significant (OR 0.76, 95% CI 0.57–1.01; *p* = 0.062; I^2^ = 94%).

Additional conservative random-effects analyses were performed for evidence subsets containing few studies (Table S7). Among the three randomized trials contributing to incident delirium, REML with Hartung–Knapp adjustment yielded a pooled OR of 0.41 (95% CI 0.09–1.86; *p* = 0.126; I^2^ = 58.1%; τ^2^ = 0.213), with a 95% prediction interval of 0.03–4.96. Thus, although the point estimate was unchanged, statistical uncertainty increased substantially under the more conservative inference method.

**Figure 3:**
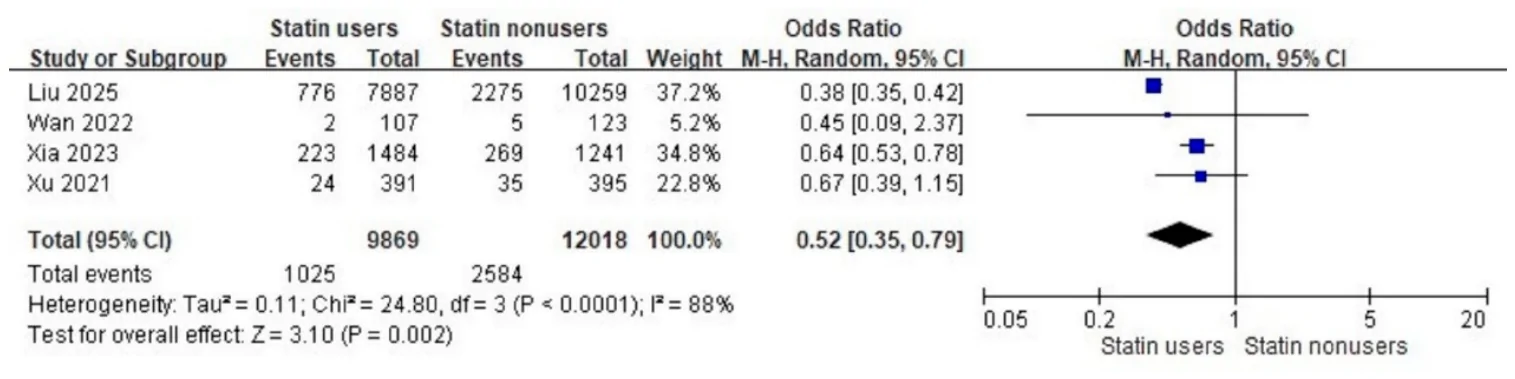
Forest plot of the odds ratios for 30-day all-cause mortality among statin users and nonusers [11,12,13,14,30].

In four studies comprising 21,687 participants that reported short-term mortality, statin use was associated with lower 30-day mortality under the primary DerSimonian–Laird model (OR 0.52, 95% CI 0.35–0.79; *p* = 0.002; I^2^ = 88%; Figure 3). Under REML with Hartung–Knapp adjustment, the point estimate remained similar, but the confidence interval widened (OR 0.52, 95% CI 0.32–0.84; *p* = 0.022; I^2^ = 84.3%; τ^2^ = 0.084). The corresponding 95% prediction interval was 0.18–1.47 and crossed the null value (Table S7). These findings indicate substantial uncertainty in the effect that might be observed across different clinical settings despite the pooled estimate remaining below unity. No significant association was observed for delirium-free days (standardized mean difference 0.25, 95% CI −0.40 to 0.91; *p* = 0.45; I^2^ = 95%; Figure S2).

### 3.4. Subgroup Analysis

**Figure 4:**
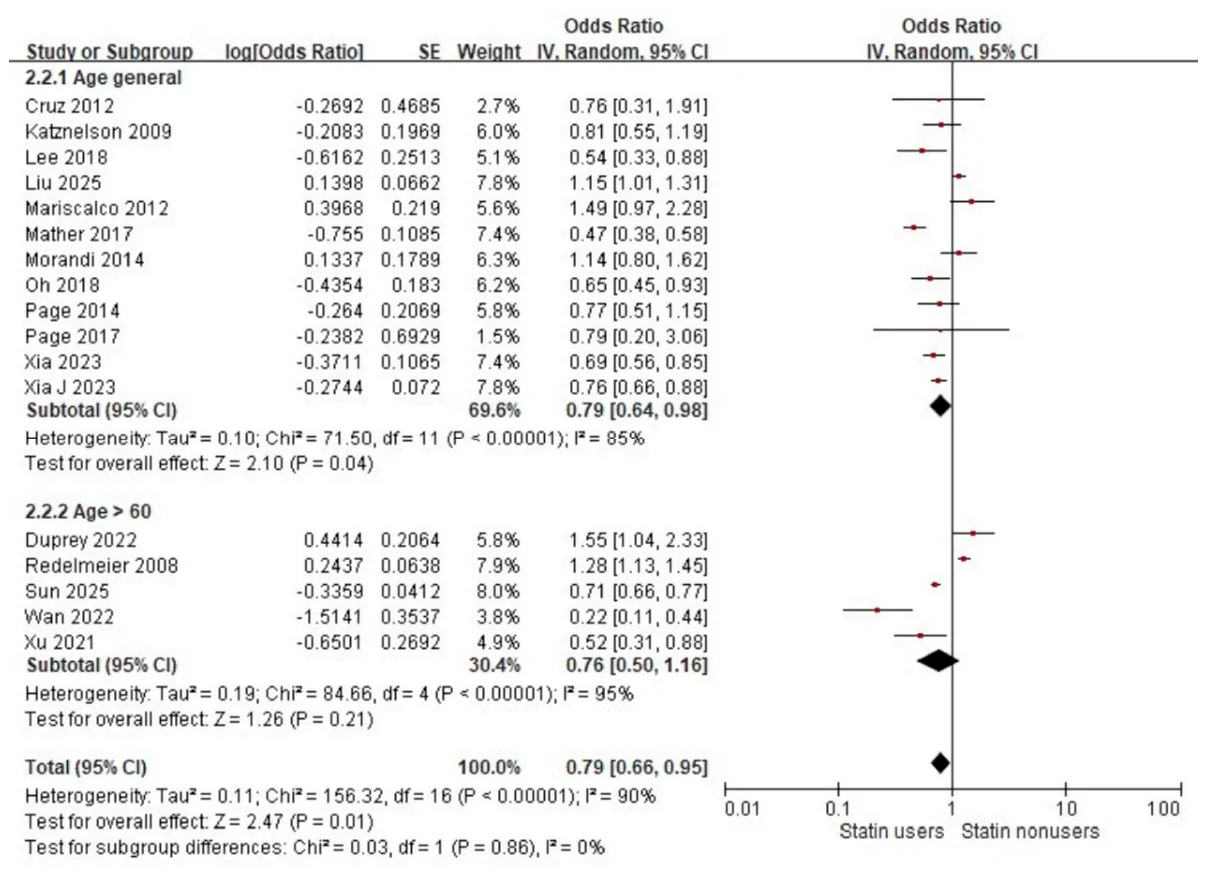
Forest plot of the summary odds ratios for delirium stratified by study-level mean or median age [10,11,12,13,14,18,19,20,21,22,23,25,26,27,28,30,31].

In the study-level age subgroup analysis, studies with a mean or median participant age below 60 years showed a statistically significant association (OR 0.79, 95% CI 0.64–0.98; *p* = 0.04; I^2^ = 85%), whereas studies enrolling older populations did not (OR 0.76, 95% CI 0.50–1.16; *p* = 0.21; I^2^ = 95%; Figure 4). The test for subgroup differences was not significant (*p* = 0.86), indicating no evidence that the pooled estimates differed between age groups.

**Figure 5:**
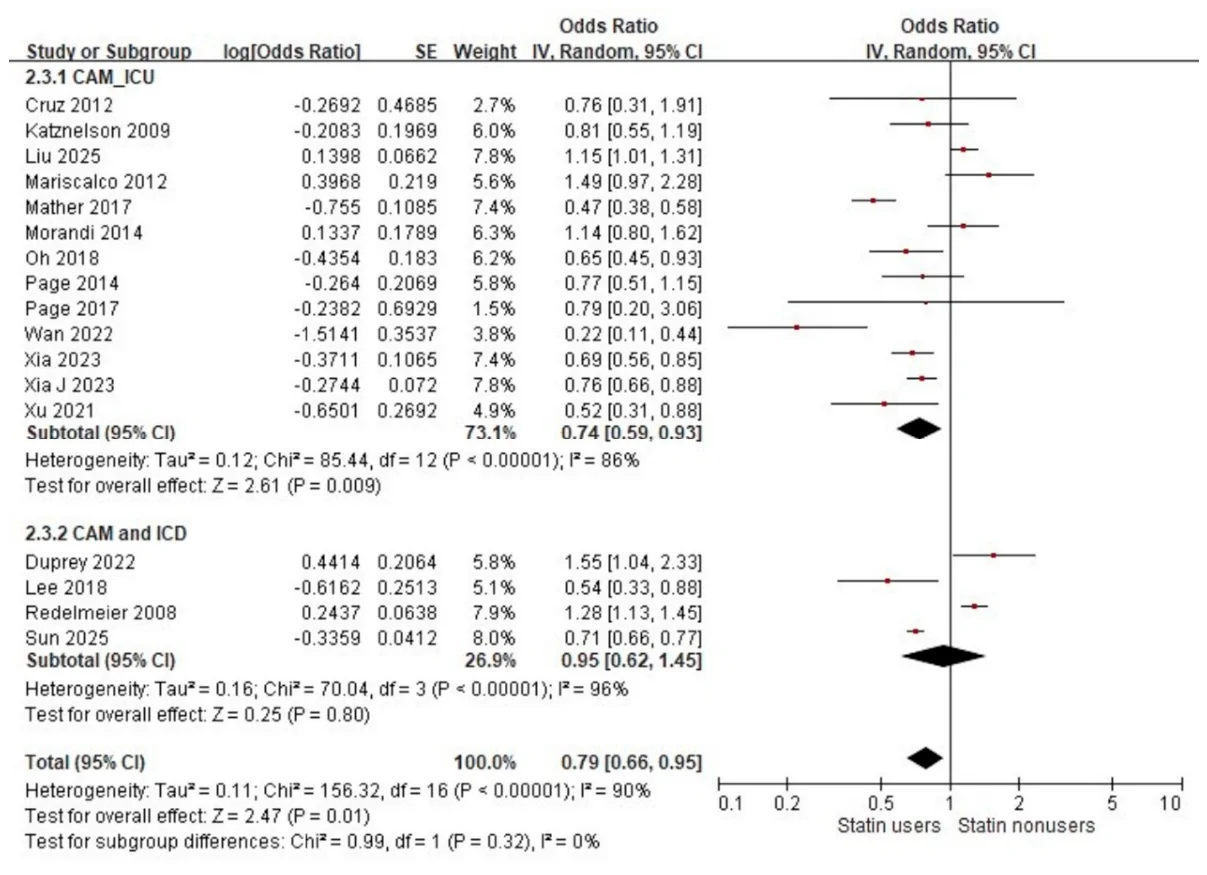
Forest plot of the summary odds ratios for delirium stratified by ascertainment method [10,11,12,13,14,18,19,20,21,22,23,25,26,27,28,30,31].

When studies were stratified by delirium ascertainment method, those using CAM-ICU showed a lower summary OR (OR 0.74, 95% CI 0.59–0.93; *p* = 0.009; I^2^ = 86%), whereas studies using CAM or ICD codes did not (OR 0.95, 95% CI 0.62–1.45; *p* = 0.80; I^2^ = 96%; Figure 5). However, the test for subgroup differences was not significant (*p* = 0.32), and residual heterogeneity remained substantial in both groups.

**Figure 6:**
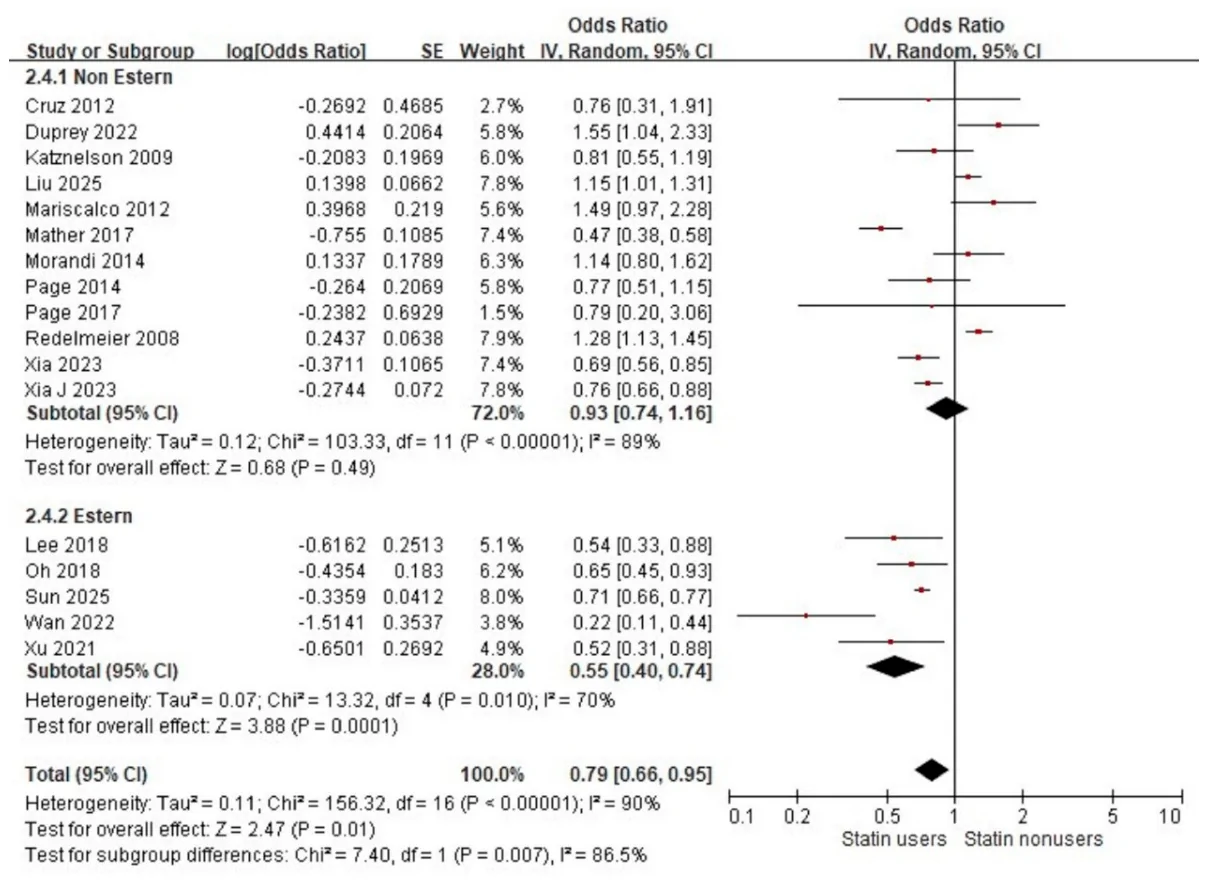
Forest plot of the summary odds ratios for delirium stratified by geographic region [10,11,12,13,14,18,19,20,21,22,23,25,26,27,28,30,31].

In the geographic subgroup analysis, studies conducted in Asian countries showed a lower summary OR (OR 0.55, 95% CI 0.40–0.74; *p* = 0.001; I^2^ = 70%), whereas studies conducted outside Asia did not (OR 0.93, 95% CI 0.74–1.16; *p* = 0.49; I^2^ = 89%; Figure 6). The subgroup difference was statistically significant (*p* = 0.007), but this aggregate-level finding should be considered exploratory because geography may be correlated with clinical setting, exposure definition, patient characteristics, and study design.

Given the substantial methodological differences between randomized and observational evidence, study design was considered a key source of heterogeneity. When studies were stratified by design, randomized trials yielded a lower pooled point estimate for delirium (OR 0.41, 95% CI 0.20–0.82; *p* = 0.01; I^2^ = 58%), whereas the pooled association among cohort and other observational studies was not statistically significant (OR 0.86, 95% CI 0.71–1.03; *p* = 0.11; I^2^ = 91%; Figure S3). The test for subgroup differences was significant (*p* = 0.04), indicating that the pooled estimates differed according to study design. An exploratory OR-only subgroup analysis was also conducted according to statin exposure timing among studies with clearly classifiable exposure definitions. Seven studies evaluating pre-existing or preoperative statin use yielded a pooled OR of 0.94 (95% CI 0.67–1.33; *p* = 0.679; I^2^ = 86.3%), whereas three studies involving newly initiated or protocol-assigned statin therapy yielded a pooled OR of 0.41 (95% CI 0.09–1.86; *p* = 0.126; I^2^ = 58.1%). The subgroup difference was statistically significant (*p* for interaction = 0.035), although substantial residual heterogeneity remained (I^2^ = 84.4%). Because exposure timing was closely correlated with study design and only three studies contributed to the newly initiated/protocol-assigned subgroup, this finding should be considered exploratory (Table S6).

### 3.5. Publication Bias

**Figure 7:**
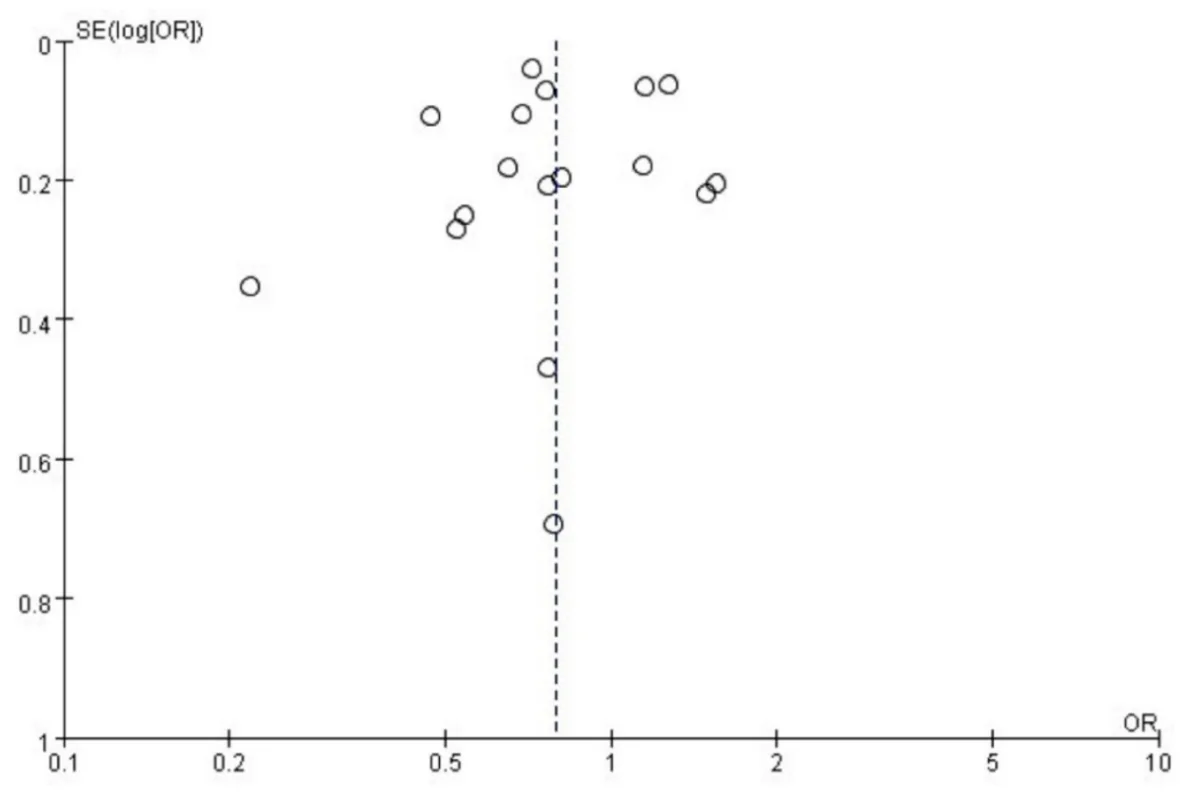
Funnel plot of studies investigating the association between statin use and delirium.

Visual inspection of the funnel plot in Figure 7 suggested an approximately symmetrical distribution. Egger’s regression test (*p* = 0.457) and Begg’s rank correlation test (*p* = 0.836) did not detect statistically significant small-study effects. However, Duval and Tweedie’s trim-and-fill procedure imputed three potentially missing studies on the right side and yielded an adjusted pooled OR of approximately 0.80 (95% CI 0.72–0.88). These findings should be interpreted cautiously because funnel-plot and trim-and-fill methods may be unreliable in the presence of substantial between-study heterogeneity.

### 3.6. Certainty of Evidence

For delirium incidence, the certainty of evidence was judged to be moderate in the prespecified GRADE assessment, with concerns related to substantial inconsistency and the predominance of nonrandomized evidence. For 30-day all-cause mortality, certainty was rated low because only a small number of studies contributed data and residual confounding could not be excluded. Certainty for delirium-free days was also rated low because few studies reported this outcome, confidence intervals were wide, and heterogeneity was considerable. These ratings should be interpreted in light of the mixed study designs and variation in exposure definitions across the evidence base.

## 4. Discussion

This updated meta-analysis found substantial heterogeneity in the association between statin use and delirium. Estimates differed according to study design: randomized trials yielded a lower pooled point estimate, whereas the pooled association among observational studies was not statistically significant. However, the apparent association in the randomized-trial subgroup was sensitive to the method of random-effects inference. When REML estimation with Hartung–Knapp adjustment was applied to the three randomized trials, the point estimate remained unchanged, but the confidence interval widened substantially and included the null. The combined estimate should therefore be regarded as exploratory rather than as a unified causal effect. Additional sensitivity analyses produced similar point estimates, but statistical uncertainty increased under more conservative analytic assumptions, including REML–Hartung–Knapp inference and restriction to explicitly multivariable-adjusted observational ORs. The pooled estimate was also sensitive to individual studies; notably, exclusion of Wan et al. attenuated the association to borderline statistical significance, while substantial heterogeneity persisted. Together, these findings indicate that although the direction of several pooled estimates was relatively consistent, their statistical precision and causal interpretation remain limited.

Our findings should be interpreted in the context of previous systematic reviews. Vallabhajosyula et al. (2017) and Chang et al. (2023) both reported pooled estimates near unity and did not identify a clear association between statin use and delirium [8,9]. The present review incorporated six additional studies published between 2022 and 2025, several of which involved large ICU or perioperative cohorts. These newer studies shifted the exploratory combined estimate below unity, but the difference between randomized and observational evidence, the high residual heterogeneity, and the sensitivity of statistical significance to analytic assumptions argue against interpreting the updated result as evidence of a definitive or consistent association. Individual study findings were also discordant. Redelmeier et al. reported higher odds of postoperative delirium among statin users [18], whereas Duprey et al. reported an effect estimate above unity [31]. These contrasting findings further underscore the substantial clinical and methodological heterogeneity across studies, including differences in patient populations, perioperative context, statin exposure definitions, delirium ascertainment, and confounder adjustment. Accordingly, the pooled estimate should not be interpreted as evidence of a consistent association across all clinical settings.

For 30-day mortality, statin exposure was associated with lower mortality in a limited subset of four studies comprising 21,687 participants. The pooled point estimate remained similar under REML with Hartung–Knapp adjustment (OR 0.52, 95% CI 0.32–0.84), suggesting that the direction of the summary association was not materially altered by the alternative random-effects approach. However, heterogeneity remained substantial (I^2^ = 84.3%), and the 95% prediction interval was wide (0.18–1.47) and crossed the null value. Thus, although the pooled association remained statistically significant, the effect expected in an individual future setting remains uncertain. Given the small evidence base and potential residual confounding, this finding should be interpreted strictly as an association rather than as evidence that statins improve short-term survival. For delirium-free days, no statistically significant association was observed (SMD 0.25, 95% CI −0.40 to 0.91), and heterogeneity was considerable (I^2^ = 95%).

The subgroup findings also warrant restraint. Given the small number of contributing studies and substantial between-study variability, the available evidence does not demonstrate a consistent association between statin exposure and delirium-free duration. Although a statistically significant within-subgroup association was observed among studies with a mean or median age below 60 years, the age interaction test was not significant (*p* = 0.86); therefore, the data do not support an age-dependent association. Similarly, the difference by delirium ascertainment method was not statistically significant at the subgroup level. The geographic subgroup difference was statistically significant, but geography is an ecological study-level characteristic that may correlate with study design, clinical setting, exposure definition, diagnostic practice, and patient characteristics. Accordingly, the geographic analysis should be considered hypothesis-generating rather than evidence of a biological regional effect.

Exposure timing may represent another source of heterogeneity. In the exploratory OR-only analysis, estimates differed between studies evaluating pre-existing or preoperative statin use and those involving newly initiated or protocol-assigned therapy. However, this subgroup finding should not be interpreted as evidence that newly initiated statin therapy is more effective. Exposure timing was strongly associated with study design, with protocol-assigned therapy largely represented by randomized trials and pre-existing exposure predominantly represented by observational studies. Therefore, the observed subgroup difference may reflect design-related differences, residual confounding, or other study-level characteristics rather than a true effect of treatment timing.

Delirium is increasingly understood as a syndrome involving neuroinflammation, endothelial and blood–brain barrier dysfunction, altered neurotransmission, and impaired cerebral microcirculation, particularly in surgical and critically ill populations [5]. Statins have pleiotropic anti-inflammatory and vascular effects that provide biological plausibility for further investigation [32]. Nevertheless, mechanistic plausibility should not be taken as confirmation of clinical efficacy, particularly when the clinical evidence is heterogeneous and dominated by observational studies. Future randomized trials should prespecify statin type, dose, exposure timing, patient population, and follow-up duration and should use standardized delirium assessment methods.

From a clinical standpoint, the present findings should not alter established indications for statin initiation or continuation. Decisions regarding continuation of pre-existing statin therapy in hospitalized patients should remain based on cardiovascular indications and the individual clinical context rather than on the current evidence regarding delirium prevention. The available evidence does not support initiating statin therapy specifically to prevent delirium.

This review has several limitations. Most included studies were observational, limiting causal inference and introducing potential confounding by indication. The extent and quality of adjustment varied substantially across studies, and a stricter sensitivity analysis restricted to explicitly multivariable-adjusted observational ORs was not statistically significant. Because adjusted estimates were not available for all studies, the primary synthesis necessarily combined adjusted and unadjusted study-level estimates, which may have contributed to residual confounding and between-study heterogeneity. Residual confounding and reverse causation cannot be excluded. The temporal relationship between statin exposure and delirium was also inconsistent: some studies evaluated chronic or preadmission use, whereas others assessed perioperative or in-hospital exposure. Although an exploratory exposure-timing subgroup analysis suggested a difference between pre-existing/preoperative and newly initiated/protocol-assigned therapy, this analysis was based on a limited number of clearly classifiable studies and was strongly confounded by study design. Accordingly, the subgroup finding cannot establish an independent effect of exposure timing. In observational studies, treatment discontinuation or non-initiation may have been influenced by clinical deterioration, and patients who remained stable long enough to receive statins during hospitalization may have differed systematically from non-users. Interstudy heterogeneity remained substantial across sensitivity analyses, likely reflecting differences in clinical setting, baseline delirium risk, ascertainment method, statin indication, treatment timing, statin type and dose, and study design. Additional subgroup analyses according to ICU versus non-ICU setting, cardiac versus noncardiac surgery, and individual statin type could not be performed reliably because of overlapping clinical categories, sparse numbers within subgroups, and inconsistent reporting of statin-specific exposure. The corresponding 95% prediction interval was wide (0.33–1.87) and crossed the null value, further underscoring substantial between-study variability and limiting the generalizability of the pooled estimate. Detailed data on dose–response relationships and statin lipophilicity were often unavailable. The updated search was limited to PubMed, Embase, the Cochrane Library, and manual reference-list screening; trial registries, preprint servers, dedicated grey-literature sources, and citation-index databases were not systematically searched. Consequently, incomplete retrieval of unpublished or non-indexed studies cannot be excluded. Finally, formal tests did not detect statistically significant small-study effects, but trim-and-fill suggested potentially missing studies, and funnel-plot methods have limited reliability in the presence of substantial heterogeneity.

## 5. Conclusions

This updated meta-analysis suggests a possible association between statin exposure and lower delirium occurrence in some analyses, while randomized and observational evidence differed and substantial heterogeneity persisted. The exploratory combined estimate remained similar in several sensitivity analyses, but uncertainty increased with REML-Hartung–Knapp inference and the association was not statistically significant when restricted to explicitly multivariable-adjusted observational ORs. Statin use was also associated with lower 30-day mortality in a small subset of studies, but the evidence does not establish a causal survival benefit. Current findings do not support initiating statins specifically for delirium prevention. Further adequately powered randomized trials should distinguish pre-existing use from new initiation and standardize statin type, dose, exposure timing, delirium assessment, and follow-up.

## Figures and Tables

**Table 1:** Baseline characteristics of included studies.

Author and Year	Study Type	Country	Study Population	Statin Use (n)	Age (Years, Mean ± SD)	Incident Delirium: n (%)	Risk Estimate (95% CI)	30-Day All-Cause Mortality, n
Redelmeier et al. 2008 [18]	Retrospective cohort study	Canada	Patients who underwent elective surgery	Statin users 19,501	71.9 ± 4.7	273 (1.4%)	aOR 1.28 (1.12–1.46)	Not provided
				Statin nonusers 264,657	73.9 ± 6.1	2912 (1.1%)		Not provided
Katznelson et al. 2009 [19]	Prospective cohort study	Canada	Patients who underwent cardiac surgery	Statin users 686	Not provided	73 (10.6%)	Not provided	Not provided
				Statin nonusers 383		49 (12.8%)		Not provided
Cruz et al. 2012 [20]	Prospective cohort study	Brazil	Patients who underwent cardiac surgery	Statin users 68	62.9 ± 10.9	8 (11.8%)	Not provided	Not provided
				Statin nonusers 101	61.5 ± 11.4	15 (14.9%)		Not provided
Mariscalco et al. 2012 [21]	Prospective cohort study	Italy	Patients who underwent cardiac surgery	Statin users 1577	67.4 ± 9.3	53 (3.4%)	OR 1.49 (0.97–2.29)	Not provided
				Statin nonusers 1577	67.5 ± 9.2	36 (2.3%)		Not provided
Morandi et al. 2014 [22]	Prospective cohort study	The United States	Patients in the ICU	Statin users 319	65 (58–72]	253 (79.3%)	Not provided	Not provided
				Statin nonusers 444	57 (46–68]	342 (77%)		Not provided
Page et al. 2014 [23]	Prospective cohort study	The United Kingdom	Patients in the ICU	Statin users 151	77 ± 11	50 (33%)	Not provided	2 ± 2
				Statin nonusers 319	63 ± 19	125 (40%)		3 ± 2
Needham et al. 2016 [24]	Randomized controlled trial	The United States	Patients who underwent surgery	Rosuvastatin 40 mg/day 137	52 ± 18	Not provided	Not provided	4.76 ± 3
				Placebo 135	52 ± 16	Not provided		4.03 ± 2.61
Mather et al. 2017 [25]	Retrospective cohort study	United States	Patients in the ICU	Statin users 1475	<55: 21.3% 55–70: 37.8% >70: 40.9%	Not provided	aOR 0.47 (0.38–0.56)	Not provided
				Statin nonusers 1475	<55: 20.7% 55–70: 33.5% >70: 43.8%	Not provided		Not provided
Page et al. 2017 [26]	Randomized controlled trial	The United Kingdom	Patients receiving mechanical ventilation in the ICU	Simvastatin 80 mg/day 71	61.9 ± 15.3	66 (93%)	Not provided	5.7 ± 5.1
				Placebo 71	62.1 ± 17.3	67 (94%)		6.1 ± 5.2
Oh et al. 2018 [27]	Retrospective cohort study	South Korea	Patients who underwent total knee arthroplasty under spinal anesthesia	Statin users 922	71.5 ± 6.6	35 (3.5%)	aOR 0.66 (0.45–0.97)	Not provided
				Statin nonusers 5028	70.9 ± 6.9	269 (5.4%)		Not provided
Lee et al. 2018 [28]	Retrospective cohort study	South Korea	Patients who underwent vascular surgery	Statin users 291	69 (61–75]	25 (8.6%)	aOR 0.54 (0.33–0.87)	Not provided
				Statin nonusers 291	70 (61–74]	39 (13.4%)		Not provided
Sohrevardi et al. 2021 [29]	Randomized controlled trial	Iran	Patients with delirium	Atorvastatin 40 mg/day 40	51 ± 19	Not provided	Not provided	2 ± 1
				Placebo 50	65 ± 24	Not provided		1 ± 0.25
Xu et al. 2021 [30]	Randomized controlled trial	China	Patients who underwent elective surgery under general anesthesia	Rosuvastatin 40 mg/day 410	66.3 ± 5.1	23 (5.6%)	aOR 0.52 (0.31–0.89)	Rosuvastatin group: 24
				Placebo 411	66.5 ± 5.3	42 (13.5%)		Placebo group: 35
Duprey et al. 2022 [31]	Prospective cohort study	The United States	Patients who underwent major noncardiac elective surgery	Statin users 323	Not provided	88 (27.2%)	RR 1.34 (0.92–1.95)	Not provided
				Statin nonusers 237	Not provided	46 (19.4%)		Not provided
Wan et al. 2022 [11]	Randomized controlled trial	China	Patients who underwent surgery for a malignant tumor	Statin users 107	69 ± 6.7	12 (11.2%)	aOR 0.22 (0.11–0.44)	2
				Placebo 123	68.3 ± 6.0	45 (36.6%)		5
Xia et al. 2023 [12]	Retrospective cohort study	The United States	Patients with chronic obstructive pulmonary disease in the ICU	Statin users 1484	73 (67–80]	199 (13.4%)	aOR 0.69 (0.56–0.85)	223
				Statin nonusers 1241	69 (61–78]	237 (19.1%)		269
Xia J et al. 2023 [13]	Retrospective cohort study	The United States	Patients with congestive heart failure in the ICU	Statin users 5446	75 (67–83]	Not provided	aOR 0.76 (0.66–0.87)	Not provided
				Statin nonusers 2950	72 (60–84]	Not provided		Not provided
Sun et al. 2025 [10]	Retrospective cohort study	Taiwan	Patients with type 2 diabetes mellitus	Statin users 55,045	79.9 ± 10.6	1034 (1.9%)	HR 0.73 (0.67–0.79)	Not provided
				Statin nonusers 55,045	78.9 ± 10.6	1436 (2.6%)		
Liu et al. 2025 [14]	Cross-sectional study	The United States	Patients receiving mechanical ventilation in the ICU	Statin users 7887	69.1 ± 11.4	624 (7.9%)	aOR 1.15 (1.01–1.37)	776
				Statin nonusers 10,259	59.0 ± 17.6	757 (7.4%)		2275

Abbreviation: aOR, adjusted odds ratio; HR, hazard ratio; ICU, intensive care unit; RR, relative risk; SD, standard deviation.

## Data Availability

The data used in this study were derived from previously published articles. All extracted data and analytic materials supporting the findings of this review are available from the corresponding author upon reasonable request.

## Supplementary Materials

The following supporting information can be downloaded at: https://www.mdpi.com/article/10.3390/biomedicines14102291/s1, Supplementary materials associated with this manuscript include Tables S1–S7 and Figures S1–S5, as cited in the main text. Table S1. The PRISMA checklist. Table S2. The quality assessment of included cohort studies. Table S3. Adjusted confounding risk factors among 14 observational studies. Table S4. Sensitivity analysis of each removed study. Table S5. Robustness and sensitivity analyses for the primary delirium outcome. Table S6. Exploratory subgroup analysis according to statin exposure timing. Table S7. Additional Sensitivity Analyses Using REML Random-Effects Models With Hartung–Knapp Adjustment. Figure S1. Risk of bias summary: review authors’ judgements about each risk of bias item for each included study. Figure S2. Forest plot of studies on delirium-free days between statin users and nonusers. Figure S3. Subgroup analysis of study design. Figure S4. The results of Duval and Tweedie’s Trim-and Fill Funnel Plot. Figure S5. Summary of sensitivity analyses for the primary delirium outcome. References [10,11,12,13,14,18,19,20,21,22,23,25,26,27,28,30,31] are cited in the Supplementary Materials.

## Appendix A

Box A1 Search strategies. PUBMED on 20 October 2025 MeSH terms 1. “Hydroxymethylglutaryl-CoA Reductase Inhibitors” = 37,051 2. “delirium” = 14,636 Text terms 3. “statin” = 73,108 4. “atorvastatin” = 12,480 5. “cerivastatin” = 814 6. “fluvastatin” = 2343 7. “lovastatin” = 13,945 8. “pravastatin” = 5324 9. “rosuvastatin” = 5265 10. “simvastatin” = 12,946 11. “delirium” = 28,617 Search strings (all inclusive): [(1 OR 3 OR 4 OR 5 OR 6 OR 7 OR 8 OR 9 OR 10) = 79,375 AND (2 OR 11) = 28,617] = 96 Published after 2022/1/1 to now = 24 Cochrane Library on 20 October 2025 MeSH terms 1. “Hydroxymethylglutaryl-CoA Reductase Inhibitors” = 5046 2. “delirium” = 1781 Text terms 3. “statin” = 8481 4. “atorvastatin” = 6389 5. “cerivastatin” = 204 6. “fluvastatin” = 782 7. “lovastatin” = 1064 8. “pravastatin” = 2127 9. “rosuvastatin” = 3146 10. “simvastatin” = 4337 11. “delirium” = 7569 Search strings (all inclusive): [(1 OR 3 OR 4 OR 5 OR 6 OR 7 OR 8 OR 9 OR 10) = 18,965 AND (2 OR 11) = 7569] = 28 Published after 2022/1/1 to now = 6 EMBASE on 20 October 2025 Emtree term 1. ‘hydroxymethylglutaryl-coa reductase inhibitors’/exp OR ‘hydroxymethylglutaryl-coa reductase inhibitors’ = 218,899 2. ‘delirium’/exp OR ‘delirium’ = 59,946 Text terms 3. “statin” = 59,377 4. “atorvastatin” = 54,083 5. “cerivastatin” = 4163 6. “fluvastatin” = 11,329 7. “lovastatin” = 6084 8. “pravastatin” = 23,239 9. “rosuvastatin” = 23,640 10. “simvastatin” = 47,818 Search strings: 3. [(1 OR 3 OR 4 OR 5 OR 6 OR 7 OR 8 OR 9 OR 10) = 232,645 AND (2)] AND (‘clinical article’/de OR ‘clinical trial’/de OR ‘cohort analysis’/de OR ‘controlled clinical trial’/de OR ‘controlled study’/de OR ‘cross sectional study’/de OR ‘major clinical study’/de OR ‘observational study’/de OR ‘prospective study’/de OR ‘randomized controlled trial’/de OR ‘randomized controlled trial topic’/de OR ‘retrospective study’/de) = 402 Published after 2022/1/1 to now = 131
