# Supplementary material for: Statin Use and the Risks of Delirium and Short-Term Mortality in Hospitalized Adults: An Updated Systematic Review and Meta-Analysis
Source: Biomedicines. 2026 Oct 9;14(10):2291. doi: 10.3390/biomedicines14102291 (PMC13701808; doi:10.3390/biomedicines14102291)
Supplement: Supplementary file 1 [file biomedicines-14-02291-s001.zip › biomedicines-4553044-supplementary.pdf]

Table S1. The PRISMA checklist

PRISMA 2020 Checklist

Section and Topic	Item #	Checklist item	Location where item is reported
**TITLE**			
Title	1	Identify the report as a systematic review.	Page 1
**ABSTRACT**			
Abstract	2	See the PRISMA 2020 for Abstracts checklist.	Page 2-3
**INTRODUCTION**			
Rationale	3	Describe the rationale for the review in the context of existing knowledge.	Page 4-5
Objectives	4	Provide an explicit statement of the objective(s) or question(s) the review addresses.	Page 5-6
**METHODS**			
Eligibility criteria	5	Specify the inclusion and exclusion criteria for the review and how studies were grouped for the syntheses.	Page 7-8
Information sources	6	Specify all databases, registers, websites, organisations, reference lists and other sources searched or consulted to identify studies. Specify the date when each source was last searched or consulted.	Page 6-7
Search strategy	7	Present the full search strategies for all databases, registers and websites, including any filters and limits used.	Page 7 and Box 1
Selection process	8	Specify the methods used to decide whether a study met the inclusion criteria of the review, including how many reviewers screened each record and each report retrieved, whether they worked independently, and if applicable, details of automation tools used in the process.	Figure 1, Page 8-9
Data collection process	9	Specify the methods used to collect data from reports, including how many reviewers collected data from each report, whether they worked independently, any processes for obtaining or confirming data from study investigators, and if applicable, details of automation tools used in the process.	Page 8-9
Data items	10a	List and define all outcomes for which data were sought. Specify whether all results that were compatible with each outcome domain in each study were sought (e.g. for all measures, time points, analyses), and if not, the methods used to decide which results to collect.	Page 8-9
	10b	List and define all other variables for which data were sought (e.g. participant and intervention characteristics, funding sources). Describe any assumptions made about any	Page 7-9

PRISMA 2020 Checklist

Section and Topic	Item #	Checklist item	Location where item is reported
		missing or unclear information.	
Study risk of bias assessment	11	Specify the methods used to assess risk of bias in the included studies, including details of the tool(s) used, how many reviewers assessed each study and whether they worked independently, and if applicable, details of automation tools used in the process.	Page 9
Effect measures	12	Specify for each outcome the effect measure(s) (e.g. risk ratio, mean difference) used in the synthesis or presentation of results.	Page 9-10
Synthesis methods	13a	Describe the processes used to decide which studies were eligible for each synthesis (e.g. tabulating the study intervention characteristics and comparing against the planned groups for each synthesis (item #5)).	Page 9-10
	13b	Describe any methods required to prepare the data for presentation or synthesis, such as handling of missing summary statistics, or data conversions.	Page 9-10
	13c	Describe any methods used to tabulate or visually display results of individual studies and syntheses.	Page 9-10
	13d	Describe any methods used to synthesize results and provide a rationale for the choice(s). If meta-analysis was performed, describe the model(s), method(s) to identify the presence and extent of statistical heterogeneity, and software package(s) used.	Page 9-10
	13e	Describe any methods used to explore possible causes of heterogeneity among study results (e.g. subgroup analysis, meta-regression).	Page 9-10
	13f	Describe any sensitivity analyses conducted to assess robustness of the synthesized results.	Page 10
Reporting bias assessment	14	Describe any methods used to assess risk of bias due to missing results in a synthesis (arising from reporting biases).	Page 10
Certainty assessment	15	Describe any methods used to assess certainty (or confidence) in the body of evidence for an outcome.	Page 10-11
**RESULTS**			
Study selection	16a	Describe the results of the search and selection process, from the number of records identified in the search to the number of studies included in the review, ideally using a flow diagram.	Page 12, figure 1
	16b	Cite studies that might appear to meet the inclusion criteria, but which were excluded, and explain why they were excluded.	Figure 1
Study characteristics	17	Cite each included study and present its characteristics.	Page 12 and table 1

PRISMA 2020 Checklist

Section and Topic	Item #	Checklist item	Location where item is reported
Risk of bias in studies	18	Present assessments of risk of bias for each included study.	Page 12-13 and figure S1, table S2
Results of individual studies	19	For all outcomes, present, for each study: (a) summary statistics for each group (where appropriate) and (b) an effect estimate and its precision (e.g. confidence/credible interval), ideally using structured tables or plots.	Page 13 and table 1
Results of syntheses	20a	For each synthesis, briefly summarise the characteristics and risk of bias among contributing studies.	Page 13 and figure S1, table S2
	20b	Present results of all statistical syntheses conducted. If meta-analysis was done, present for each the summary estimate and its precision (e.g. confidence/credible interval) and measures of statistical heterogeneity. If comparing groups, describe the direction of the effect.	Page 13, figure 2, 3
	20c	Present results of all investigations of possible causes of heterogeneity among study results.	Results; Tables S6–S7
	20d	Present results of all sensitivity analyses conducted to assess the robustness of the synthesized results.	Results; Tables S4–S7 and Figure S5
Reporting biases	21	Present assessments of risk of bias due to missing results (arising from reporting biases) for each synthesis assessed.	Page 15
Certainty of evidence	22	Present assessments of certainty (or confidence) in the body of evidence for each outcome assessed.	Page 16
**DISCUSSION**			
Discussion	23a	Provide a general interpretation of the results in the context of other evidence.	Page 17-19
	23b	Discuss any limitations of the evidence included in the review.	Page 19-20
	23c	Discuss any limitations of the review processes used.	Page 19-20
	23d	Discuss implications of the results for practice, policy, and future research.	Page 19-20
**OTHER INFORMATION**			
Registration and protocol	24a	Provide registration information for the review, including register name and registration number, or state that the review was not registered.	Page 6

PRISMA 2020 Checklist

Section and Topic	Item #	Checklist item	Location where item is reported
	24b	Indicate where the review protocol can be accessed, or state that a protocol was not prepared.	Page 6
	24c	Describe and explain any amendments to information provided at registration or in the protocol.	Page 6
Support	25	Describe sources of financial or non-financial support for the review, and the role of the funders or sponsors in the review.	Page 21
Competing interests	26	Declare any competing interests of review authors.	Page 21
Availability of data, code and other materials	27	Report which of the following are publicly available and where they can be found: template data collection forms; data extracted from included studies; data used for all analyses; analytic code; any other materials used in the review.	Page 21

From: Page MJ, McKenzie JE, Bossuyt PM, Boutron I, Hoffmann TC, Mulrow CD, et al. The PRISMA 2020 statement: an updated guideline for reporting systematic reviews. BMJ 2021;372:n71. doi: 10.1136/bmj.n71

For more information, visit: http://www.prisma-statement.org/

Figure S1 Risk of bias summary: review authors' judgements about each risk of bias item for each included study.

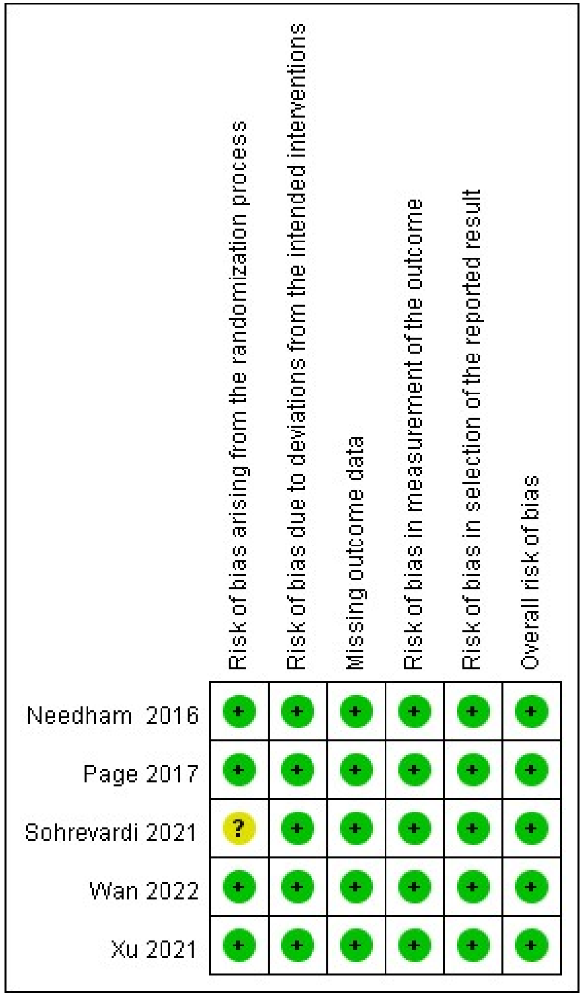

Table S2. The quality assessment of included cohort studies.

Study	Selection	Comparability	Exposure/Outcome	Quality score
Redelmeier _2008	★★★★	★★	★★	8
Katznelson_2009	★★★★	★★	★★	8
Cruz_2012	★★★★	★	★★	8
Mariscalco_2012	★★★★	★★	★★	8
Morandi_2014	★★★	★★	★★	7
Page_2014	★★★	★★	★★	7
Mather_2017	★★★	★★	★	6
Lee_2018	★★★	★★	★	6
Oh_2018	★★★	★★	★	7

Duprey_2022	★★★★	★★	★★★	9
Xia_2023	★★★	★★	★★	7
Xia J_2023	★★★	★★	★★	7
Sun_2025	★★★★	★★	★★	8
Liu_2025	★★★★	★★	★★★	9

Table S3. Adjusted confounding risk factors among 14 observational studies.

Study	Adjusted confounding risk factors
Redelmeier 2008	Adjusted for age, sex, social status, prior admissions, use of medications, type of surgery and duration of surgery.
Katznelson 2009	The author mentioned that multivariate analysis was performed; however, the adjusted variables were not presented in the study.
Cruz 2012	Adjusted for preoperative factors (age, gender, a history of stroke, peripheral vascular disease, depression, renal disease, diabetes mellitus, hypertension, acute myocardial infarction, arrhythmia, heart failure and anemia) and intraoperative factors (valve replacement and myocardial revascularization).
Mariscalco 2012	Adjusted for age, body surface area, emergency, cardiogenic shock, preoperative left ventricular ejection fraction classes, hypertension, dyslipidemia, chronic obstructive pulmonary disease, peripheral vascular disease, history of cerebrovascular accident, estimated glomerular filtration rate, medication uses, type of operation, cardiopulmonary bypass time, aortic cross-clamp time, postoperative intraaortic balloon pump need, inotropic support, blood transfusion, fresh frozen plasma transfusion, platelets transfusion, postoperative atrial fibrillation, postoperative acute kidney injury.
Morandi 2014	Adjusted for age, gender, history of stroke, ischemic heart disease, chronic heart failure, cancer, liver disease, renal disease, chronic obstructive pulmonary disease, diabetes, peripheral vascular disease, psychiatric illness, depression, Framingham Stroke Risk Profile, Charlson Comorbidity Index, current cigarette smoking and alcohol abuse, activities of daily living, baseline cognitive impairment, medication use, insurance status, race, weight, and years of education.
Page 2014	Adjusted for C-reactive protein

Mather 2017	Adjusted for age, length of stay, male, race, comorbidities, Deyo-Charlson Comorbidity index
Oh 2018	All variables of $p < 0.1$ in univariable logistic regression analysis were included in multivariable logistic regression model. However, the author did not list the adjusted variables.
Lee 2018	Adjusted for operative time, type of surgery, emergency, general anesthesia, hypertension, diabetes mellitus, chronic obstructive pulmonary disease, chronic renal failure, heart failure, anemia, age, smoking alcohol, preoperative beta-blocker
Duprey 2022	Adjusted for age, sex, surgery type, Charlson comorbidity index, APACHE II score, SOFA score, baseline cognitive function, and worst postoperative pain.
Xia_2023	Adjusted for age, respiratory rate, heart rate, diastolic blood pressure, oxygen saturation, Richmond Agitation-Sedation Scale score, sepsis, length of ICU stay, use of vasopressors (vasopressin, norepinephrine, epinephrine), β-blockers, antiemetics, antibiotics, and other baseline characteristics (such as Simplified Acute Physiology Score II and Charlson Comorbidity Index).
Xia J_2023	Adjustments were made for dozens of variables, including age, gender, vital signs, Simplified Acute Physiology Score II, Charlson Comorbidity Index, laboratory parameters (such as hematocrit, hemoglobin, white blood cell count, platelet count, blood urea nitrogen, creatinine, and blood glucose), comorbidities (e.g., myocardial infarction, coronary artery disease, cancer), and medication use (β-blockers, ACE inhibitors/ARBs, diuretics, and vasopressors).
Sun_2025	Adjusted age group, sex, income level, degree of urbanization, duration of type 2 diabetes mellitus, follow-up period, types of antidiabetic medications (e.g., metformin, sulfonylureas, DPP-4 inhibitors), adapted diabetes complication severity index, Charlson comorbidity index, and multiple comorbidities (such as nephropathy, cerebrovascular disease, and congestive heart failure). A multivariable Cox regression model was further adjusted for the PSM variables and competing risks.

Liu_2025	Adjusted for age, sex, body mass index, Acute Physiology Score III, SOFA score, sepsis, duration of mechanical ventilation, duration of continuous renal replacement therapy, length of hospital stay, multiple chronic comorbidities (such as coronary artery disease, congestive heart failure, renal failure, and diabetes mellitus), and medication use (such as sedatives/analgesics and vasopressors).

Table S4. Sensitivity analysis of each removed study.

Studies	ORs	Lower CI	Upper CI	I^2 (%)	P-value
Overall	0.79	0.66	0.95	90	0.01
Omitting					
Cruz 2012	0.79	0.66	0.96	90	0.02
Duprey 2022	0.76	0.63	0.92	90	0.004
Katznelson 2009	0.79	0.65	0.96	90	0.02
Lee 2018	0.81	0.67	0.98	90	0.03
Liu 2025	0.77	0.64	0.93	88	0.007
Mariscalco 2012	0.77	0.63	0.92	90	0.005
Mather 2017	0.83	0.69	0.99	88	0.04
Morandi 2014	0.77	0.64	0.94	90	0.009
Oh 2018	0.80	0.66	0.97	90	0.03
Page 2014	0.80	0.66	0.96	90	0.02
Page 2017	0.79	0.66	0.96	90	0.01
Redelmeier 2008	0.77	0.64	0.91	86	0.003
Sun 2025	0.80	0.65	0.98	89	0.03
Wan 2022	0.84	0.70	1.00	89	0.05

Xia 2023	0.80	0.66	0.97	90	0.03
Xia J 2023	0.79	0.65	0.97	90	0.03
Xu 2021	0.81	0.67	0.98	90	0.03

Study or Subgroup	Statin users Mean	SD	Total	Statin nonusers Mean	SD	Total	Weight	Std. Mean Difference IV, Random, 95% CI
Needham 2016	4.76	3	137	4.03	2.61	135	25.7%	0.26 [0.02, 0.50]
Page 2014	2	2	151	3	2	369	26.0%	-0.50 [-0.69, -0.31]
Page 2017	5.7	5.1	71	6.1	5.2	71	24.9%	-0.08 [-0.41, 0.25]
Sohrevardi 2021	2	1	40	1	0.25	50	23.4%	1.43 [0.97, 1.90]
**Total (95% CI)**			**399**			**625**	**100.0%**	**0.25 [-0.40, 0.91]**

Heterogeneity: $Tau^2 = 0.42$; $Chi^2 = 66.21$, $df = 3$ ($P < 0.00001$); $I^2 = 95\%$
Test for overall effect: $Z = 0.76$ ($P = 0.45$)

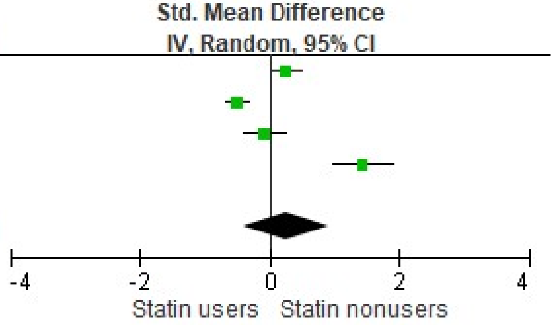

Figure S2. Forest plot of studies on delirium-free days between statin users and nonusers.

Study or Subgroup	log[Odds Ratio]	SE	Weight	Odds Ratio IV, Random, 95% CI
**2.1.1 RCT**				
Page 2017	-0.2382	0.6929	1.5%	0.79 [0.20, 3.06]
Wan 2022	-1.5141	0.3537	3.8%	0.22 [0.11, 0.44]
Xu 2021	-0.6501	0.2692	4.9%	0.52 [0.31, 0.88]
**Subtotal (95% CI)**			**10.1%**	**0.41 [0.20, 0.82]**
Heterogeneity: Tau² = 0.21; Chi² = 4.79, df = 2 (P = 0.09); I² = 58%				
Test for overall effect: Z = 2.52 (P = 0.01)				
**2.1.2 Cohort or observational study**				
Cruz 2012	-0.2692	0.4685	2.7%	0.76 [0.31, 1.91]
Duprey 2022	0.4414	0.2064	5.8%	1.55 [1.04, 2.33]
Katznelson 2009	-0.2083	0.1969	6.0%	0.81 [0.55, 1.19]
Lee 2018	-0.6162	0.2513	5.1%	0.54 [0.33, 0.88]
Liu 2025	0.1398	0.0662	7.8%	1.15 [1.01, 1.31]
Mariscalco 2012	0.3968	0.219	5.6%	1.49 [0.97, 2.28]
Mather 2017	-0.755	0.1085	7.4%	0.47 [0.38, 0.58]
Morandi 2014	0.1337	0.1789	6.3%	1.14 [0.80, 1.62]
Oh 2018	-0.4354	0.183	6.2%	0.65 [0.45, 0.93]
Page 2014	-0.264	0.2069	5.8%	0.77 [0.51, 1.15]
Redelmeier 2008	0.2437	0.0638	7.9%	1.28 [1.13, 1.45]
Sun 2025	-0.3359	0.0412	8.0%	0.71 [0.66, 0.77]
Xia 2023	-0.3711	0.1065	7.4%	0.69 [0.56, 0.85]
Xia J 2023	-0.2744	0.072	7.8%	0.76 [0.66, 0.88]
**Subtotal (95% CI)**			**89.9%**	**0.86 [0.71, 1.03]**
Heterogeneity: Tau² = 0.10; Chi² = 138.79, df = 13 (P < 0.00001); I² = 91%				
Test for overall effect: Z = 1.62 (P = 0.11)				
**Total (95% CI)**			**100.0%**	**0.79 [0.66, 0.95]**
Heterogeneity: Tau² = 0.11; Chi² = 156.32, df = 16 (P < 0.00001); I² = 90%				
Test for overall effect: Z = 2.47 (P = 0.01)				
Test for subgroup differences: Chi² = 4.07, df = 1 (P = 0.04), I² = 75.5%				

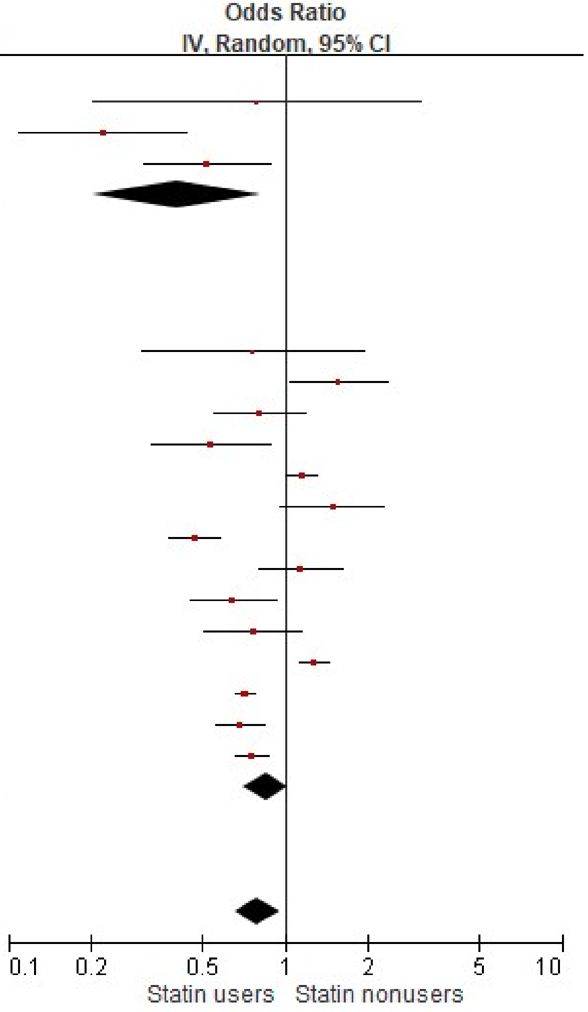

Figure S3. Subgroup analysis of study design.

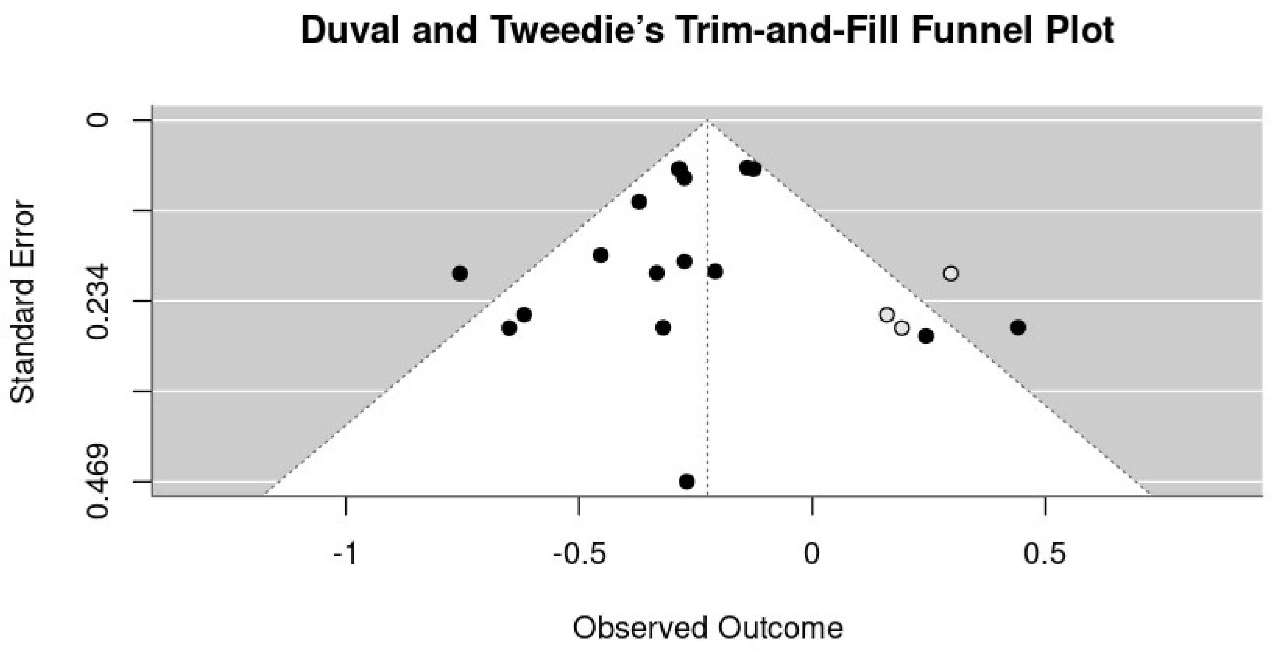

Figure S4. The results of Duval and Tweedie's Trim-and Fill Funnel Plot.

Estimated number of missing studies on the right side: 3 (SE = 2.7990)

Random-Effects Model (k = 20; tau^2 estimator: REML)

logLik	deviance	AIC	BIC	AICc
-3.7210	7.4419	11.4419	13.3308	12.1919

tau^2 (estimated amount of total heterogeneity): 0.0241 (SE = 0.0152)
tau (square root of estimated tau^2 value): 0.1551
I^2 (total heterogeneity / total variability): 63.36%
H^2 (total variability / sampling variability): 2.73

Test for Heterogeneity:
Q(df = 19) = 43.5119, p-val = 0.0011

Model Results:

estimate	se	zval	pval	ci.lb	ci.ub	
-0.2249	0.0511	-4.3984	<.0001	-0.3251	-0.1247	***

Signif. codes: 0 ‘***’ 0.001 ‘**’ 0.01 ‘*’ 0.05 ‘.’ 0.1 ‘ ’ 1

Table S5. Robustness and sensitivity analyses for the primary delirium outcome.

**Analysis**	**No. of studies**	**Restriction/model**	**Pooled OR (95% CI)**	**p value**	**I^2 (%)**
Original exploratory analysis	17	DerSimonian-Laird random-effects	0.79 (0.66-0.95)	0.01	90
OR-only analysis	15	Excluded Duprey 2022 (RR) and Sun 2025 (HR)	0.76 (0.61-0.94)	0.01	89
Excluding cross-sectional study	16	Excluded Liu 2025	0.77 (0.64-0.93)	0.007	88
Alternative random-effects inference	17	REML with Hartung-Knapp adjustment	0.79 (0.62-1.00)	0.047	92.7
Adjusted observational ORs only	7	Observational studies explicitly reporting multivariable-adjusted ORs	0.76 (0.57-1.01)	0.062	94

Note: The combined analysis of different relative effect measures was regarded as exploratory. The adjusted observational analysis was restricted to studies explicitly reporting multivariable-adjusted odds ratios with sufficient information on covariate adjustment.

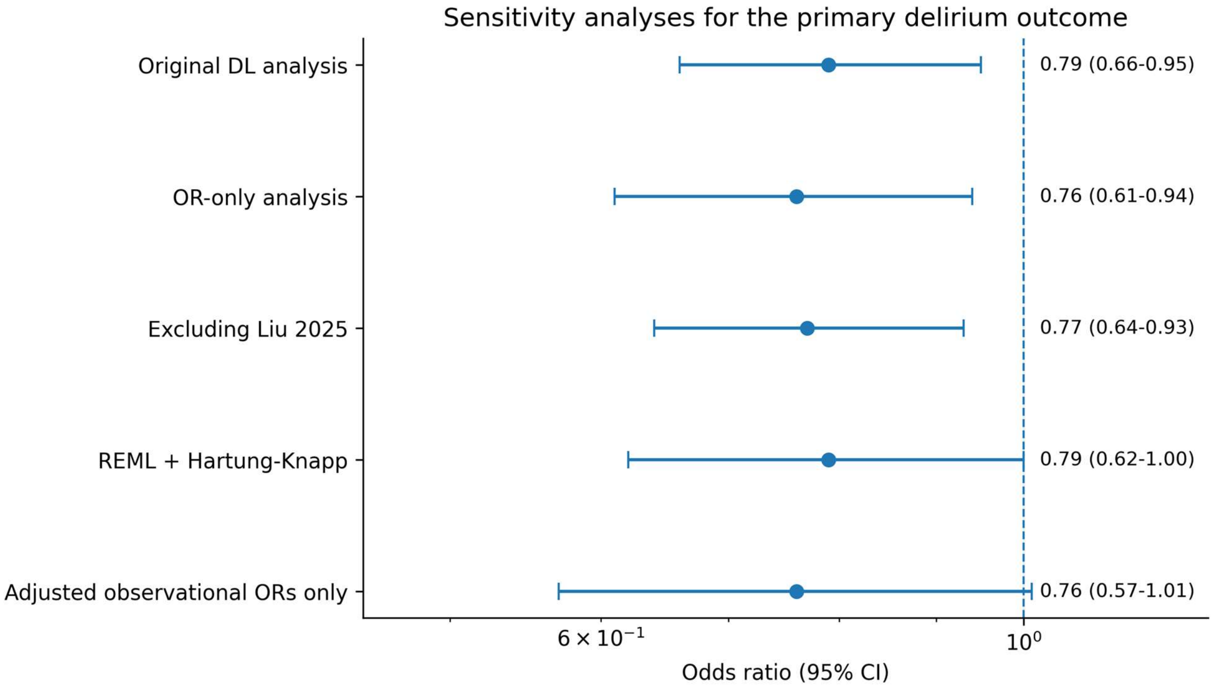

Point estimates and 95% confidence intervals are shown for the original DerSimonian–Laird analysis and additional robustness and sensitivity analyses.

Figure S5. Summary of sensitivity analyses for the primary delirium outcome.

Table S6. Exploratory subgroup analysis according to statin exposure timing

Exposure timing	No. of studies	Pooled OR (95% CI)	p value	I^2 (%)	95% PI	p for subgroup difference
Pre-existing / preoperative	7	0.94 (0.67–1.33)	0.679	86.3	0.40–2.21	
Newly initiated / protocol-assigned	3	0.41 (0.09–1.86)	0.126	58.1	0.03–4.96	**0.035**

Analyses were restricted to OR-reporting studies with clearly classifiable statin exposure timing and were performed using REML random-effects models with Hartung–Knapp adjustment. Studies with mixed or unclear exposure timing were excluded. The subgroup comparison should be considered exploratory because exposure timing was closely correlated with study design.

Table S7. Additional Sensitivity Analyses Using REML Random-Effects Models With Hartung–Knapp Adjustment

Outcome/analysis	No. of studies	Model	Pooled OR (95% CI)	p value	I^2 (%)	τ^2	95% prediction interval
Incident delirium, randomized controlled trials only	3	REML with Hartung–Knapp adjustment	0.41 (0.09–1.86)	0.126	58.1	0.213	0.03–4.96
30-day all-cause mortality	4	REML with Hartung–Knapp adjustment	0.52 (0.32–0.84)	0.022	84.3	0.084	0.18–1.47

Note: CI, confidence interval; OR, odds ratio; PI, prediction interval; REML, restricted maximum likelihood. Hartung–Knapp adjustment was applied to account for uncertainty in the estimated between-study variance, particularly in analyses with a small number of contributing studies. The wide prediction intervals, both of which crossed the null value of 1.0, indicate substantial uncertainty regarding the true effect that might be observed in a future comparable study.
